# Melatonin dose and timing: Do we have it right?

**DOI:** 10.1017/S109285292510062X

**Published:** 2025-10-23

**Authors:** Shobha George, Anushree Sripathy, Aysha Rehman, Joseph George, Kanishk Chirayil, Emma Frost, Nalini Ramanathan, Simy Joseph, Karim Ghobrial-Sedky, Akshay Sripathy, Jose Maldonado, Jeff McCagh, Maju Mathew Koola

**Affiliations:** 1 College of Arts and Sciences, University of Delaware, Newark, DE, USA; 2 Stony Brook University, Stony Brook, NY, USA; 3 Departments of Psychiatry and Human Behavior and Child and Adolescent Psychiatry, The Children’s Hospital of Philadelphia, Philadelphia, PA, USA; 4 School for Science and Mathematics, South Carolina Governor’s Hartsville, SC, USA; 5 Stone Bridge High School, Ashburn, VA, USA; 6 Cooper Neurological Institute, Cooper University Health Care, Camden, NJ, USA; 7 Family Medical Group, St Peter’s Health Partners, Albany, NY, USA; 8 Department of Medicine, Teamhealth Inc, Knoxville, TN, USA; 9 Department of Psychiatry, Family Health Centers of San Diego, San Diego, CA, USA; 10 Guilderland High School, Hamlet, NY, USA; 11 Department of Psychiatry and Behavioral Sciences, Stanford University School of Medicine, Stanford, CA, USA; 12 Sheppard Pratt Health System, Baltimore, MD, USA; 13 Department of Psychiatry and Behavioral Sciences, George Washington University, Washington, DC, USA

**Keywords:** circadian rhythm, delirium, insomnia, melatonin, ramelteon

## Abstract

Melatonin is an easily accessible, widely used drug for sleep issues, disrupted sleep–wake cycles, and jet lag, available in a variety of forms and dosages. Melatonin is also used in hospital settings to promote sleep onset, particularly in elderly patients, as a circadian rhythm regulator. Despite the popularity of melatonin, it is not approved by the US Food and Drug Administration (FDA). This creates ambiguity surrounding its proper usage for optimum results, including dosage and time of administration. The objective of this article is to shed light on the best timing to administer melatonin. Melatonin is a hormone that our body naturally produces to regulate our biological clock. Even though our body has a built-in “sleep system,” many people still suffer from chronic sleep disorders such as insomnia. Melatonin has also proved to help prevent delirium in hospitalized patients due to its circadian rhythm regulatory effects. The elderly are at risk of developing insomnia because as one ages, melatonin production decreases. The most convenient solution for insomnia is to take melatonin supplements. To optimize the effects of melatonin supplements, proper dosage and timing must be considered. Additionally, patients who are oppositional to bedtime, which is known as bedtime resistance, are typically more willing to go to bed following melatonin administration. Melatonin administration at around 6 PM (1–2 hours before bedtime) is optimal to regulate sleep cycles of patients, and it can help with bedtime resistance. This should be the standard of care in all hospitals, nursing homes, and at home.

Melatonin is a hormone that the body naturally produces and is secreted in the pineal gland. The amino acid tryptophan is first hydroxylated and then decarboxylated into serotonin.^1^ Enzymes from the pineal gland then turn serotonin into melatonin. After it is synthesized, melatonin gets released into the body’s circulation to become effective. There are different types of melatonin receptors: MT1, MT2, and MT3, where MT1 and MT2 have a higher affinity than MT3.^2^ These receptors are found throughout the body in various organs and vessels. Melatonin is the core of the biological clock, as it regulates the circadian rhythm, allowing one to differentiate between day and night. Darkness stimulates the production of melatonin, whereas light prevents it. Systematic reviews and meta-analyses have shown that melatonin can effectively treat insomnia.^3^ ^,^^4^

## Best timing to administer melatonin

The objective of this article is not to explore melatonin usage simply for sedation properties, but to explore using it to regulate sleep rhythm and hygiene. The theory is that taking it earlier will also help regulate the cycle for patients to fall asleep at a more “normal” time. Additionally, patients who are oppositional to bedtime, which is known as bedtime resistance, are typically more willing to go to bed following melatonin administration. Melatonin administration around 6 PM^5^ is optimal to regulate sleep cycles of patients, and it can help with bedtime resistance.

In many hospitals, when melatonin administration is ordered, the default timing is 9 PM or 10 PM. Administration of medications at 6 PM (neither evening nor bedtime) is not typical. Hence, this would be an inconvenience for nurses, which is why hospitals are reluctant to change their policies. Although this change in timing poses an additional task for nurses, the default timing for melatonin administration may be changed in electronic medical records to optimize sleep for patients who face bedtime resistance.

The variety of melatonin forms and dosages poses difficulty for patients choosing what is best for them. It has been proven that low doses of melatonin are effective for treating insomnia in patients because they best mimic a normal circadian rhythm.^5^ Doses of 0.3 mg and 1 mg were observed to stimulate psychological levels like those in young adults during the night.^5^ A review of 16 studies conducted with adults over the age of 55 years concluded that higher doses of melatonin lead to higher levels in the body for a longer time, which leaves room for undesired effects such as daytime drowsiness. Those suffering from insomnia are unable to get the rest that they need. Moreover, with age progression, natural peak nocturnal melatonin concentrations decrease.^6^ Melatonin given around 6:00 PM proved to decrease sleep latency significantly in elderly patients.^5^ Although 6:00 PM seems relatively early to take melatonin, patients taking it during the early evening shift their sleep earlier, which increases the total amount of sleep time.^6^ This means that the time taken to fall asleep is reduced, providing additional hours of sleep formerly consumed by insomnia. For optimal results, aim to take melatonin roughly 3–4 hours before your desired sleep time. Therefore, if you aim to be asleep by 10 or 11 PM, taking it around 6 or 7 PM would align with this recommendation (GoodRx).

## Dosing

Since melatonin is not approved by the FDA, there are no true maximum doses as there would be for an FDA-approved drug. Clinically, it has been used upwards of 20 mg but is typically prescribed in doses of 3 mg, 6 mg, 9 mg, and 12 mg (package insert).^7^ There are not enough data to administer more than 12 mg while also titrating other psychoactive drugs.

Due to an absence of FDA regulation, the actual concentration of the drug compared to what is indicated on the package insert becomes closely scrutinized. A study of 31 melatonin supplements determined that the real concentration of melatonin varied between −83% and + 478% of its labeled concentration.^8^ This unreliability in melatonin concentrations makes dosing difficult, not to mention possibly affecting research studies. Additionally, melatonin’s bioavailability has a broad range of 1%–74% due to the dose and development of the specific supplement.^8^ Despite these hurdles, melatonin has proved to be an effective sleep aid. Common side effects of melatonin include headache and drowsiness (1 in 10 people, package insert).^7^

## Melatonin for delirium

Hospitalized patients face the risk of developing a major cognitive disorder known as delirium, characterized by conscious states of confusion and circadian rhythm disruptions.^9^ Evidence shows that since chronic sleep deprivation is a major stressor for the body, it can lead to delirium, especially due to symptoms such as high blood pressure and increased levels of cortisol and proinflammatory cytokines.^10^ Additionally, chronic sleep deprivation creates sleep debt, which can also be another causal factor of delirium.^10^ This can be difficult to manage, as patients with symptoms tend to be uncooperative. Research has shown that melatonin can be an 9effective treatment for preventing delirium and potentially reducing the duration of delirium-related episodes. Urinary 6-sulfatoxymelatonin is the main metabolite of melatonin and was found to be lower in patients when they were in a phase of hyperactive delirium, whereas patients who had hypoactive delirium showed higher levels of 6-sulfatoxymelatonin.^10^ Eighteen studies with a total of 2137 hospitalized patients concluded that melatonin did indeed contribute to the prevention of delirium due to the regulation of patients’ sleep patterns.^9^

## Ramelteon

Since melatonin is a dietary supplement, it is not approved by the FDA but is still the primary recommended drug for insomnia by the American Academy of Family Physicians.^6^ Additional medication that can be used to treat insomnia includes ramelteon, which, contrary to melatonin, is FDA-approved. Ramelteon is a melatonin receptor agonist, specifically for MT1 and MT2 receptors.^11^ Contrary to melatonin, ramelteon is absorbed by the body much quicker due to its high affinity for the melatonin receptors; hence, it is administered 30 minutes before bedtime. There is only one dose for ramelteon, which is 8 mg (package insert).^12^

## Conclusion and future directions

The best time to administer melatonin is around 6 PM (1–2 hours before bedtime). This should be the standard of care in all hospitals and nursing homes and at home. There should be a change in practice and hospital policies.

## Data Availability

Data from this manuscript can be found in the articles listed under “References.”
